# Human umbilical cord blood-mesenchymal stem cell-derived secretome in combination with atorvastatin enhances endothelial progenitor cells proliferation and migration

**DOI:** 10.12688/f1000research.23547.1

**Published:** 2020-06-05

**Authors:** Yudi Her Oktaviono, Suryo Ardi Hutomo, Makhyan Jibril Al-Farabi, Angliana Chouw, Ferry Sandra

**Affiliations:** 1 Department of Cardiology and Vascular Medicine, Faculty of Medicine, Universitas Airlangga, Soetomo General Academic Hospital, Surabaya, Indonesia; 2 Stem Cell Division, Prodia Laboratory, Jakarta, Indonesia; 3 Department of Biochemistry and Molecular Biology, Faculty of Dentistry, Universitas Trisakti, Jakarta, Indonesia

**Keywords:** coronary artery disease, endothelial progenitor cells, mesenchymal stem cells, secretome, statins

## Abstract

**Background:** Human umbilical cord blood-mesenchymal stem cell (hUCB-MSC)-derived secretome is known to be able to promote neovascularization and angiogenesis, so it is also thought to have a capability to modulate endothelial progenitor cell (EPC) functions. Atorvastatin is the cornerstone of coronary artery disease (CAD) treatment which can enhance EPCs proliferation and migration. This study aims to analyze the effect of the hUCB-MSC-derived secretome and its combination with atorvastatin toward EPCs proliferation and migration.

**Methods:** EPCs were isolated from a CAD patient’s peripheral blood. Cultured EPCs were divided into a control group and treatment group of 2.5 µM atorvastatin, hUCB-MSC-derived secretome (2%, 10%, and 20% concentration) and its combination. EPCs proliferation was evaluated using an MTT cell proliferation assay, and EPC migration was evaluated using a Transwell migration assay kit.

**Results:** This research showed that hUCB-MSC-derived secretomes significantly increase EPC proliferation and migration in a dose-dependent manner. The high concentration of hUCB-MSC-derived secretome were shown to be superior to atorvastatin in inducing EPC proliferation and migration (p<0.001). A combination of the hUCB-MSC-derived secretome and atorvastatin shown to improve EPCs proliferation and migration compared to hUCB-MSC-derived secretome treatment or atorvastatin alone (p<0.001).

**Conclusions:** This study concluded that the hUCB-MSC-derived secretome work synergistically with atorvastatin treatment in improving EPCs proliferation and migration.

## Introduction

Coronary artery disease (CAD) is the leading cause of mortality and morbidity worldwide ^1^. It is responsible for the deaths of 7.2 million people or 12.2% of total deaths per year worldwide. Despite advancement in CAD management (e.g. novel antiplatelet therapy, coronary stents, percutaneous coronary intervention techniques and devices, and coronary artery bypass surgery), there are some clinical subsets of CAD which remain untreatable such as ischemic cardiomyopathy, refractory angina, and patient which cannot undergo revascularization due to clinical and anatomical complexity ^2, 3^.

It is already known that CAD is caused by atherosclerosis, which is followed by reduced levels of circulating endothelial progenitor cells (EPCs) ^4^. EPCs can differentiate into mature endothelial cells and also promote endothelial repair. Hence, increasing circulating EPC levels is proven to improve endothelial function ^5^. EPCs also had a critical role in the stimulation of angiogenesis and vasculogenesis. Hence, increasing EPC proliferation and migration may reduce ischemia and improve myocardial performance ^4, 6^.

Regenerative treatment for CAD using stem cells has been extensively studied in the last decade ^7^. However, these treatments faced challenges of low engraftment, poor survival, and low differentiation of the transplanted cells. Despite regenerative treatment shown to be promising *in vitro*, clinical studies showed unsatisfying results ^8^. Hence, the researcher started to shift regenerative treatment from cell based-treatment into cell-free treatment using paracrine stimulation ^9^. Nowadays, the usage of cell-free therapeutics as a regenerative therapy in cardiovascular diseases also started to be emerged ^9^.

The secretome is the wide array variety of paracrine factors produced by mesenchymal stem cells (MSCs). Human umbilical cord blood mesenchymal stem cells (hUCB-MSCs) derived secretome was proven could promote neovascularization, angiogenesis ^10– 13^ and improved cardiac systolic function by protecting myocardial cells from apoptosis ^14^. However, using this approach to improve neovascularization is yet to be investigated. Hence, it is hypothesized that increasing EPCs proliferation and migration by the hUCB-MSC-derived secretome may be responsible for this effect. Statins, through their pleiotropic effect, are the cornerstone of CAD treatment. Atorvastatin is one of the most prescribed statins, whose ability to modulate EPCs proliferation and migration has already been well studied in both laboratory and clinical settings ^15– 17^. Furthermore, this study aims to compare the effect of the hUCB-MSC-derived secretome, atorvastatin and the two in combination in modulating EPC proliferation and migration.

## Methods

### Sample collection

A 50–100 mL peripheral blood sample was obtained from a patient with CAD. The patient was recruited from the outpatient cardiovascular clinic at Pusat Pelayanan Jantung Terpadu, Dr. Soetomo General Hospital, Surabaya, in March 2020. The inclusion criteria were as follows: male, aged 40–59 years old, history of chronic ischemic heart disease as proven by coronary angiography results that showed >50% stenosis of left main coronary artery or >70% of other coronary arteries. The exclusion criteria were as follows: a history of percutaneous coronary intervention procedures or coronary artery bypass grafting surgery, acute coronary syndromes, and anemia.

This study protocol has an ethical clearance from the Health Research Ethics Committee of Dr. Soetomo General Hospital, Surabaya (No.1567/KEPK/X/2019, approved on 8 October 2019). The included subjects provided written informed consent before subject recruitment. All details which include personal information were omitted.

### HUCB-MSCs-derived secretome preparation

The HUCB-MSCs-derived secretome was prepared according to the previous study ^18^. HUCB-MSCs (3H Biomedical AB, Uppsala, Sweden) was cultured in MesenCult ^TM^ MSC Basal medium, supplemented with MesenCult ^TM^ Stimulatory supplement (StemCell Technologies Inc., Vancouver, Canada), and also added with penicillin and streptomycin. Upon reaching 80% confluency, the media was replaced with MesenCult ^TM^ MSC Basal medium (supplement-free media) and incubated for 24 hours. The media was collected and centrifuged. The supernatant was used as a conditioned medium that contained hUCB-MSCs-derived secretome.

### Isolation and culture of EPCs

Peripheral blood mononuclear cells (PBMCs) were isolated by density centrifugation of CAD patient’s peripheral blood using Histopaque-1077 (Sigma-Aldrich, USA). After centrifugation of peripheral blood, PBMCs then cultured with STEMLINE-II hematopoietic stem cell expansion medium (Sigma-Aldrich, USA) supplemented with stem cell factor, thrombopoietin, Flt-3 ligand, vascular endothelial growth factor, and interleukin-6. A total of 5×10 ^6^ mononuclear cells/ml were seeded into fibronectin-coated 6-well plate dish and cultured at 37°C and 5% CO _2_ levels for 5 days. Non-adherent cells were then transferred for the proliferation and migration assay. After five days of culture, EPCs were confirmed using FITC-labeled anti-human CD34 antibody (animal source was mouse, 5 μL antibody was diluted at 500 μL per 1 × 10 ^6^ cells; catalog number 60013FI, Gene ID: 947, by StemCell Technologies Inc., Vancouver, Canada) staining and examined with immunofluorescence microscopy.

### Treatment of EPCs

Cultured EPCs were divided into eight treatment groups for each proliferation and migration assays. Those treatment include control group, 2.5 µM atorvastatin, low (2%), medium (10%) and high (20%) doses of hUCB-MSC-derived secretome, and combination of 2.5 µM atorvastatin with each dose of the hUCB-MSC-derived secretome. There were n=5 replications made from each treatment.

### EPCs proliferation assay

The MTT cell proliferation assay kit (Sigma-Aldrich, St Louis, MO, USA) was used to measure EPCs proliferation as described previously ^19^. Treated EPCs were added with MTT reagent and incubated in a 37°C incubator with 5% CO _2_ for 4 hours. Proliferation was determined from the reduction of tetrazolium (MTT) into insoluble formazan product by viable EPCs mitochondria. Absorbance was measured with a microplate reader at 595 nm wavelength. EPCs proliferation was measured as optical density (OD). MTT assay was measured at day 3 after reagent addition.

### EPCs migration assay

EPCs migration was evaluated using the 24-mm diameter insert, 3-µm pore size, 6-well Transwell migration assay kit (Corning, USA). A total of 5×10 ^5^ cultured EPCs were placed in the upper part of the Transwell migration assay kit. Next, 2 mL of EPC media and each treatment were added in the lower chamber compartment and then incubated for 24 hours at 37°C. Non-migratory cells were removed manually. On the receiver plate, the new basal medium was placed and added 500 μL of trypsin + EDTA solution 0.5%, followed by 10 minutes incubation. Then, cells on the bottom surface of the membrane were stained with Giemsa and cell images were obtained on a light microscope and counted manually in n=5 random fields/sample.

### Statistical analysis

Statistical analyses were conducted using SPSS Statistics 23.0 to detect significance level at p<0.05. One-way ANOVA was used to compare groups, with Fisher’s least significant difference (LSD) post hoc test. Kruskal-Wallis test was used if there are violations to the assumption of normality and the assumption of homogeneity of variance. Correlation between variables was obtained using Spearman’s correlation followed by a linear regression test.

## Results

### Baseline characteristics and demography of CAD patient

Clinical examination, blood sampling, electrocardiography, echocardiography and coronary angiography was conducted and evaluated in order to examine the inclusion and exclusion criteria. Our sample had a 1-year history of coronary artery disease, he suffered from refractory chest pain despite the optimum medical therapy. The coronary angiography showed complex lesion (three-vessel disease with chronic total occlusion) which was not amenable to undergo revascularization. The baseline characteristics of the patient are presented in Table 1.

**Table 1.** Characteristics of the patient.

Variables	Result
Sex	Male
Age	59 years old
Body Mass Index (BMI)	27.3
Blood pressure	140/90
Heart rate	90 beats per minute
Electrocardiography	Sinus rhythm, pathological Q-waves at V-V6 leads.
Laboratory	
Total cholesterol (mg/dL)	240
Triglyseride (mg/dL)	131
LDL (mg/dL)	140
HDL (mg/dL)	55
Hemoglobin (mg/dL)	14.2
Blood glucose (mg/dL)	142
Echocardiography	
Left ventricle ejection fraction	41% (teich); 36% (biplane)
Left ventricle internal diameter	5.8 cm
Wall motion	Hypokinesia at anterior, anteroseptal, inferoseptal, other segments kinetic was normal
Valves	mild mitral regurgitation
Coronary Angiography	
Left main coronary artery (LMCA)	normal
Left anterior descending artery (LAD)	70% stenosis at osteal, chronic total occlusion (CTO) at distal
Left circumflex coronary artery (LCX)	70% stenosis at proximal, CTO at distal, grade 2 collaterals from LCX to RCA
Right coronary artery (RCA)	CTO at proximal

### EPC characteristics

**Figure 1.**
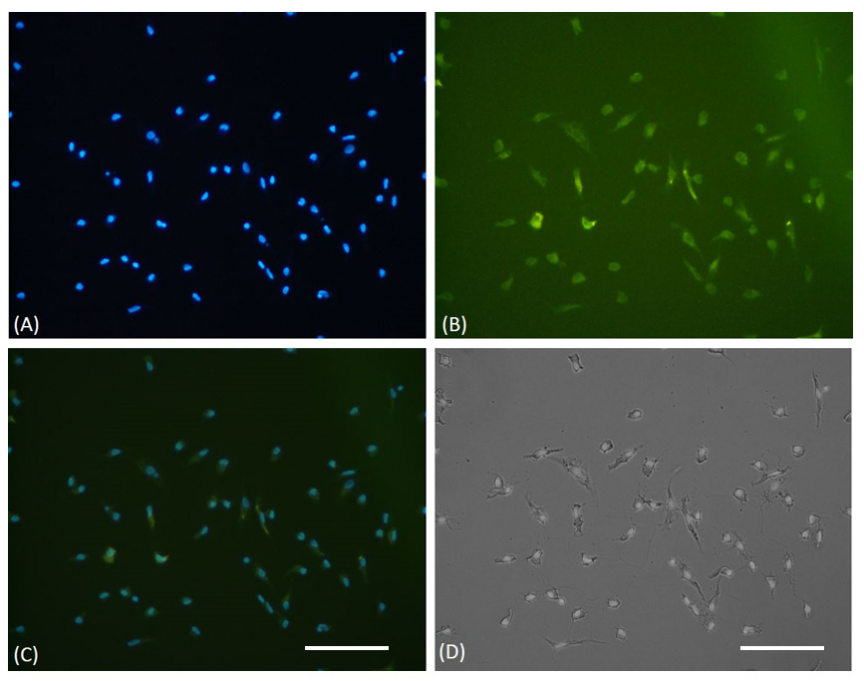
Immunofluorescence characterization of cultured EPCs. ( **A**) DAPI staining of cultured EPCs showed viable cells through blue fluorescent of cells nuclei. ( **B**) EPCs were confirmed, using FITC-labeled anti-human CD34 expression on immunofluorescence microscope. ( **C**) Merged view of DAPI and FITC stained cells. ( **D**) The light microscope view showed the spindle shape of EPCs. The white bar represents 50 µm.

EPCs were succesfully isolated and cultured from the CAD patient’s peripheral blood. It was confirmed through light microscopy and an immunofluorescence assay ( Figure 1). Raw images are available as *Underlying data* ^20^.

### HUCB-MSCs-derived secretome and atorvastatin increase EPCs proliferation

**Figure 2.**
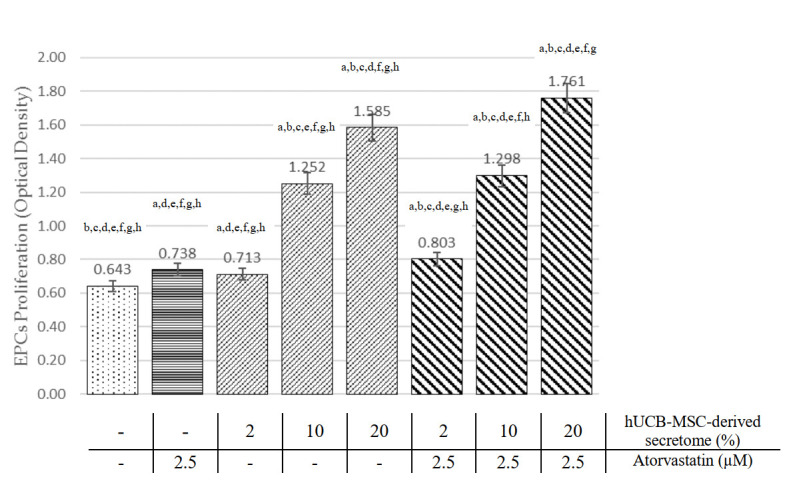
Comparison of EPC proliferation effects among all treatment groups (see text). ^a^Significant difference compared to the control group (p < 0.001). ^b^Significant difference compared to the 2.5 µM atorvastatin group (p < 0.001). ^c^Significant difference compared to the 2% hUCB-MSC-derived secretome group, (p <0.001). ^d^Significant difference compared to the 10% hUCB-MSC-derived secretome group (p < 0.001). ^e^Significant difference compared to the 20% hUCB-MSC-derived secretome group (p < 0.001), ^f^Significant difference compared to the combination of 2% hUCB-MSC-derived secretome and atorvastatin group, (p < 0.001), ^g^Significant difference compared to the combination of 10% hUCB-MSC-derived secretome and atorvastatin group, (p < 0.001). ^h^Significant difference compared to the combination of 20% hUCB-MSC-derived secretome and atorvastatin group, (p < 0.001)

EPCs were evaluated using the MTT proliferation assay. As shown in Figure 2, both atorvastatin and hUCB-MSCs-derived secretome treatment groups at all doses increase EPCs proliferation compared to the control (p<0.05, ANOVA). hUCB-MSC-derived secretome treatment showed a dose-dependent relationship with EPCs proliferation. At medium (10%) and high (20%) doses, hUCB-MSC-derived secretome was shown to elicit superior EPC proliferation than atorvastatin (OD 1.252±0.104 and 1.585±0.029, respectively, vs 0.738±0.025; p<0.01). Raw absorbance data for MTT assays are available as *Underlying data* ^20^.

Pearson’s correlation showed a significant and strong correlation between hUCB-MSCs-derived secretome treatment with EPC proliferation (r=0.954; p<0.001). The linear regression test showed an R ^2^ of 0.910.

### Combination of hUCB-MSCs-derived secretome and atorvastatin increase EPCs proliferation compared with single treatment

Figure 2 showed the combination of atorvastatin and hUCB-MSC-derived secretome at the dose of 2%, 10% and 20% concentration have significantly higher EPCs proliferation compared to atorvastatin alone (OD 0.803±0.046, 1.298±0.075 and 1.761±0.419 vs 0.738±0.025, p<0.05). In addition, combination of hUCB-MSC-derived secretome at dose of 2%, 10% and 20% with atorvastatin showed higher EPCs proliferation compared to hUCB-MSC-derived secretome alone (OD 0.803±0.046 vs 0.713±0.049, 1.298±0.075 vs 1.252±0.104 and 1.761±0.419 vs 1.585±0.029, p<0.05). The combination group showed a significant and very strong correlation with EPC proliferation (r=0.973; p<0.001), Linear regression test showed R ^2^ of 0.947.

### HUCB-MSCs-derived secretome and atorvastatin increase EPCs migration

**Figure 3.**
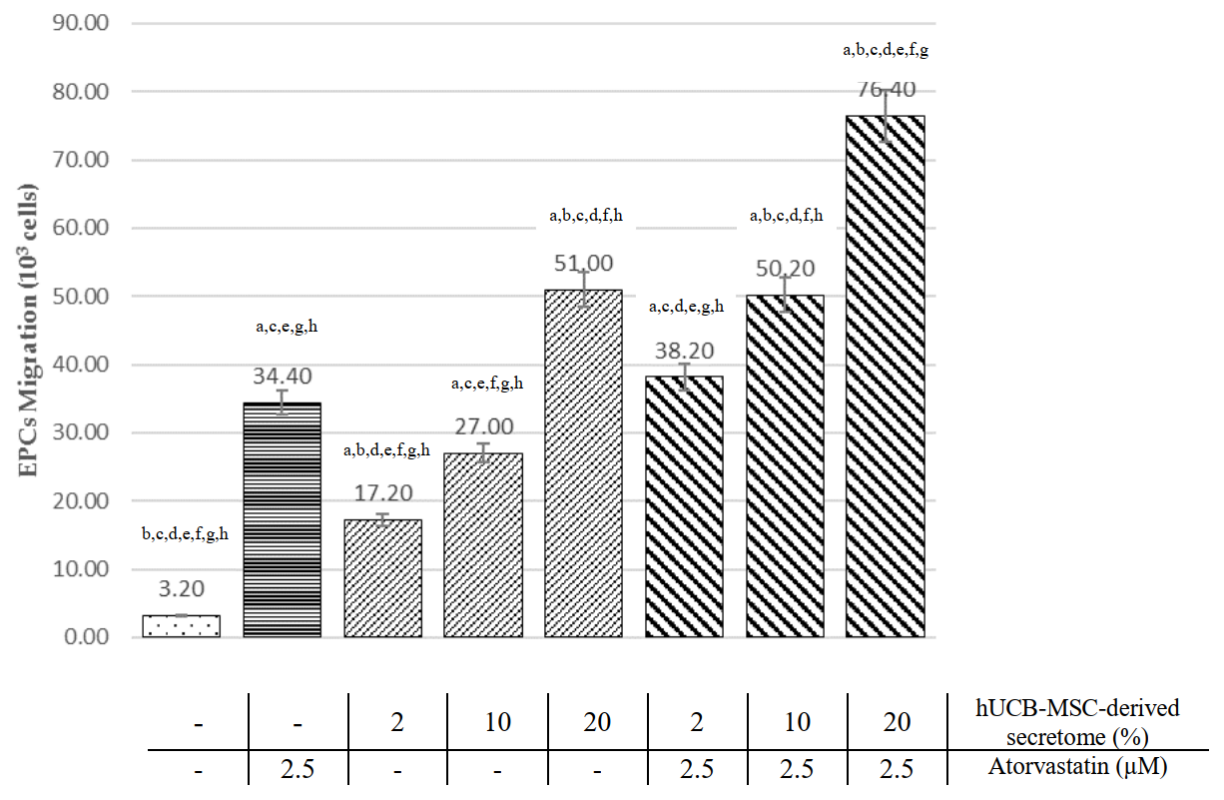
Comparison of EPCs migration effects among all treatment groups (see text). ^a^Significant difference compared to the control group (p < 0.001). ^b^Significant difference compared to the 2.5 µM atorvastatin group (p < 0.001). ^c^Significant difference compared to the 2% hUCB-MSC-derived secretome group (p < 0.001). ^d^Significant difference compared to the 10% hUCB-MSC-derived secretome group (p < 0.001). ^e^Significant difference compared to the 20% hUCB-MSC-derived secretome group (p < 0.001). ^f^Significant difference compared to the combination of 2% hUCB-MSC-derived secretome and atorvastatin group, (p < 0.001). ^g^Significant difference compared to the combination of 10% hUCB-MSC-derived secretome and atorvastatin group, (p < 0.001). ^h^Significant difference compared to the combination of 20% hUCB-MSC-derived secretome and atorvastatin group, (p < 0.001)

EPCs migration from each treatment group was analyzed using the Transwell migration assay. As shown in Figure 3, EPC treatment with atorvastatin and all doses of hUCB-MSCs-derived secretome significantly increase EPC migration compared to the control group (p<0.05, ANOVA). Treatment with 2.5 µM atorvastatin has significantly higher EPCs migration than low (2%) and medium (10%) doses of hUCB-MSC-derived secretome (34.40±3.05 vs 17.20±1.92 and 27.00±4.00, p<0.05). However, high doses (20%) of hUCB-MSC-derived secretome showed significantly higher migrated EPCs than atorvastatin (51.00±5.15 vs 34.40±3.05, p<0.001). Raw cell counts used to assess migration are available as *Underlying data* ^20^.

 Pearson’s correlation showed a significant and very strong correlation between hUCB-MSCs-derived secretome treatment with EPC migration (r=0.968; p<0.001). The linear regression test showed an R ^2^ of 0.937.

### Combination of hUCB-MSCs-derived secretome and atorvastatin increase EPCs migration compared with single treatment

In Figure 3, EPCs migration was significantly higher in combination treatment groups (atorvastatin and hUCB-MSC-derived secretome) at 2%, 10%, and 20% doses compared to the atorvastatin alone (38.20±3.49, 50.20±5.31 and 76.40±7.50 vs 34.40±3.05, p<0.001).

Combination of hUCB-MSC-derived secretome with atorvastatin also showed higher EPCs migration than the hUCB-MSC-derived secretome-only group at 2%, 10% and 20% concentrations (38.3±3.49 vs 17.20±1.92, 50.20±5.31 vs 27.00±4.00 and 76.40±7.50 vs 51.00±5.15, respectively; all p<0.001). The combination of high-dose (20%) hUCB-MSC-derived secretome and atorvastatin had the highest number of migrated EPC (76.4±7.50 × 10 ^3^ cells). The combination group had a significant and very strong correlation with EPC migration (r=0.970; p<0.001). The linear regression test showed an R ^2^ of 0.942.

## Discussion

This research showed that treatment with hUCB-MSC-derived secretome, atorvastatin and a combination of the two increased the proliferation and migration of EPCs (isolated from CAD patient’s peripheral blood). HUCB-MSC-derived secretome enhances EPCs proliferation and migration in a dose-dependent manner. The combination of hUCB-MSC-derived secretome and atorvastatin was shown to be superior to atorvastatin or hUCB-MSC-derived secretome alone.

In this research, hUCB-MSC-derived secretome treatment increased EPC proliferation in a dose-dependent manner, with the concentrations of 10% and 20% shown to be superior to atorvastatin. Previous studies showed that atorvastatin treatment is superior to other statins at improving EPC proliferation ^21– 23^. The HUCB-MSC-derived secretome is also composed of cytokines, chemokines, growth factors, proteins, and extracellular vesicles which may be involved in EPCs proliferation and migration ^13, 24^. Vascular endothelial growth factor (VEGF), stromal-derived factor-1 (SDF-1), insulin-like growth factor (IGF-1) are contained in the hUCB-MSC-derived secretome which may be involved in increasing EPC proliferation ^25^. VEGF has been shown to improve the proliferation and differentiation of EPCs through activation of Ras signaling, and the MAPK/ERK pathway ^26– 28^. SDF-1 and IGF 1 also been shown to increase the EPCs proliferation in response to the PI3K/protein kinase B signaling pathway and promote angiogenesis ^29– 31^. Hence, it is suggested that hUCB-MSC-derived secretome treatment is beneficial to improve EPC proliferation which may involve MAPK/ERK and PI3K/protein kinase B pathway.

HUCB-MSC-derived secretome treatment shown to increase EPCs migration in a dose-dependent manner, with a concentration of 20% shown to be superior to atorvastatin. Similarly, the previous study showed secretome-derived from placental-MSCs is able to significantly increase EPCs migration ^32^. The HUCB-MSC-derived secretome contains pro-angiogenic factors, such as human angiopoietin-1 (Ang-1), hepatocyte growth factor (HGF), insulin-like growth factor I (IGF-I), prostaglandin E2 (PGE2), transforming growth factor-beta 1 (TGF-ß1), vascular cell adhesion protein 1 (VCAM-1) and vascular endothelial growth factor (VEGF) ^33^. MSCs also have immunomodulatory and anti-inflammatory properties, as it contributes to the maintenance of self-renewal capacity through E-Prostanoid 2 (EP2) ^34^ and immune cell activation and maturation, including CD4 ^+^ helper T cells, B cells, dendritic cells, natural killer cells, monocytes and macrophages ^35^. The HUCB-MSC-derived secretome also has a higher anti-inflammatory effect than other MSCs ^36^ and antioxidant properties, as proven in previous studies conducted in renal injury ^37^ and ischemic stroke ^38^. Inflammatory stimuli and oxidative stress are also known to impair EPCs migration ^39^. Chemoattractant gradient was an important driving factor to induce EPCs mobilization. Hence, a high concentration of growth factors in the HUCB-MSC-derived secretome increase the gradient between the top and lower parts of the Transwell migration assay may lead into an increase of EPCs migration. taken together, wide array molecules and multiple possible pathways involved in HUCB-MSCs secretome treatment seem to be responsible for its superiority against atorvastatin.

The synergistic effect of the HUCB-MSC-derived secretome with atorvastatin in enhancing EPCs proliferation and migration was demonstrated in this study. These combinations significantly increase EPCs proliferation and migratory activity by up to two-fold. Previously, The combination of MSCs with another compound, including statins, was shown to have beneficial effects in angiogenesis and neovascularization ^40– 42^. Co-culture of MSCs and EPCs have been shown to demonstrate improved EPC proliferation and migration, and enhance their angiogenic capacity ^43, 44^. However, the exact mechanism of these combinations to improve EPCs proliferation and migration is yet to be investigated. It is speculated that the involvement of multiple pathways may be responsible for its superiority against HUCB-MSCs-derived secretome or atorvastatin alone.

A mitogen-activated protein kinase (MAPK) pathway has been known to play a role in increasing EPCs proliferation ^45^. Cell cycle progression through increased Cyclin D1 expression mediated by PI3K/Akt and MAPK pathway also involved in EPCs proliferation ^46^. While increasing microRNA 221/222 expression shown to reduce EPCs proliferation capabilities ^47^. HUCB-MSC-derived secretome treatment was speculated to improve EPCs proliferation through MAPK/ERK and PI3K/protein kinase B pathway. While atorvastatin improves EPCs proliferation through downregulation of microRNA 221/222 expression ^47^. The involvement of these multiple pathways may result in higher EPCs proliferation in the combination group.

Enhanced growth factor levels through hUCB-MSC-derived secretome treatment will augment the chemoattractant gradient ^37^, thus leading to enhanced migration of EPCs. The anti-inflammatory and antioxidant properties of hUCB-MSC-derived secretome also speculated to improve EPCs migration ^35, 37^. While atorvastatin can increase the production of endothelial nitric oxide synthase and nitric oxide, which reduces the oxidative stress that impairs EPCs migration ^42^. Statin also could prevent EPCs senescence by upregulating TRF2 of EPCs, hence enhance migratory capacity ^48^. Those facts suggested that the combination of hUCB-MSC-derived secretome and atorvastatin will have superior EPCs migration through the involvement of multiple pathways.

In summary, hUCB-MSC-derived secretome may be developed and combined with atorvastatin treatment in CAD patients to improve EPCs proliferation and migration. However, this research did not measure the exact composition of the hUCB-MSC-derived secretome. The previous study showed that the secretome from another type of MSC can increase EPCs migration but not EPCs proliferation ^49^. Hence, further research should be directed to identify the substance within the hUCB-MSC-derived secretome which is responsible for increasing EPC proliferation and migration, and compare it with other MSC secretomes. Further research should also verify the multiple pathways which may be responsible for the improvement of EPCs proliferation and migration in the combination group.

## Conclusions

High dose hUCB-MSC-derived secretome outperforms atorvastatin to improve EPC proliferation and migration. A combination of hUCB-MSC-derived secretome with atorvastatin seems to be beneficial in promoting neovascularization through improved EPCs proliferation and migration effect compared to hUCB-MSC-derived secretome or atorvastatin alone.

## Data availability

### Underlying data

Figshare: Human umbilical cord blood-mesenchymal stem cell-derived secretome in combination with atorvastatin enhances endothelial progenitor cells proliferation and migration. https://doi.org/10.6084/m9.figshare.12186507.v2 ^20^.

This project contains the following underlying data:

- RAW Data - F1000 revisi2 by SAH (XLSX). (Raw absorbance data from MTT proliferation assay and cell counts from the Transwell migration assay.)
- RAW DATA f1000 (ZIP). (Raw images generated in this study, including images used to generate cell counts and raw immunofluorescence images.)

Data are available under the terms of the Creative Commons Attribution 4.0 International license (CC-BY 4.0).
