# Supplementary material for: Human umbilical cord blood-mesenchymal stem cell-derived secretome in combination with atorvastatin enhances endothelial progenitor cells proliferation and migration
Source: F1000Res. 2021 May 10;9:537. Originally published 2020 Jun 5. [Version 2] doi: 10.12688/f1000research.23547.2 (PMC8358709; doi:10.12688/f1000research.23547.2)
Supplement: Peer review history

# Peer review: Reviewer response for version 2

**DOI:** 10.5256/f1000research.56385.r85035

**Published:** 2021-08-11

**Authors:** Anwar Santoso

**Affiliations:** 1 Department of Cardiology, Faculty of Medicine, University of Indonesia, Jakarta, Indonesia; 2 National Cardiovascular Centre, Harapan Kita Hospital, Jakarta, Indonesia

I have re-reviewed this interesting manuscript on secretome and I have no further comments on that.

 Certainly, I would be happy to recommend that this wonderful paper would be indexed.

Is the work clearly and accurately presented and does it cite the current literature?

Yes

If applicable, is the statistical analysis and its interpretation appropriate?

Yes

Are all the source data underlying the results available to ensure full reproducibility?

Partly

Is the study design appropriate and is the work technically sound?

Yes

Are the conclusions drawn adequately supported by the results?

Yes

Are sufficient details of methods and analysis provided to allow replication by others?

Partly

Reviewer Expertise:

1. Cardiovascular disease (particularly ischemic heart disease) 2. Lipidology and diabetes mellitus 3. Hypertension 4. Stem cell and regenerative medicine

I confirm that I have read this submission and believe that I have an appropriate level of expertise to confirm that it is of an acceptable scientific standard.

# Peer review: Reviewer response for version 2

**DOI:** 10.5256/f1000research.56385.r85034

**Published:** 2021-05-12

**Authors:** Anwar Tandar

**Affiliations:** 1 Division of Cardiovascular Medicine, University of Utah School of Medicine, Salt Lake City, UT, USA

Concerns were appropriately addressed. Now acceptable for indexing.

Is the work clearly and accurately presented and does it cite the current literature?

Yes

If applicable, is the statistical analysis and its interpretation appropriate?

I cannot comment. A qualified statistician is required.

Are all the source data underlying the results available to ensure full reproducibility?

No

Is the study design appropriate and is the work technically sound?

Partly

Are the conclusions drawn adequately supported by the results?

Partly

Are sufficient details of methods and analysis provided to allow replication by others?

Partly

Reviewer Expertise:

Clinician, Interventional Cardiologist

I confirm that I have read this submission and believe that I have an appropriate level of expertise to confirm that it is of an acceptable scientific standard.

# Peer review: Reviewer response for version 1

**DOI:** 10.5256/f1000research.25984.r64523

**Published:** 2020-09-03

**Authors:** Anwar Tandar, John David Symmons

**Affiliations:** 1 University of Utah School of Medicine, Salt Lake City, UT, USA; 2 Division of Cardiovascular Medicine, University of Utah School of Medicine, Salt Lake City, UT, USA

This is an interesting research involving the evaluation of EPCs in CAD and association with statin.

 Several concerns should be addressed:

1. It is derived from one patient. The conclusion and validity need to be addressed seriously when there is sample from one patient. In addition, since there is only one patient, why is there even a need for inclusion and exclusion criteria
2. Typgraphical error in the Introduction... refractory angina, and patients
3. Reference needed for the statement: HUCB-MSCs-derived secretome preparation. The media was collected......(reference 3).
4. Treatment of EPCs...Please describe the rationale for 5 replications.  Please describe the rationale behind the selected number 5.4.
5. All abbreviations need to be spelled out in the beginning or first time used
6. Similar to the rational for EPCs treatment, in EPC migrations assay section: Please describe the rationale for 5 fields and describe the sizes of the field.
7. This interesting research should investigate more patients to allow a stronger interpretation and to allow more sound conclusion
8. Other statins should also be investigated as they are being used widely in real world.
9. The exact composition of the hUCB-MSc derived secretome should be described.

In summary, this research needs more diligent work especially with the number of samples from more robust patients population.

Is the work clearly and accurately presented and does it cite the current literature?

Yes

If applicable, is the statistical analysis and its interpretation appropriate?

I cannot comment. A qualified statistician is required.

Are all the source data underlying the results available to ensure full reproducibility?

No

Is the study design appropriate and is the work technically sound?

Partly

Are the conclusions drawn adequately supported by the results?

Partly

Are sufficient details of methods and analysis provided to allow replication by others?

Partly

Reviewer Expertise:

Anwar Tandar, MD: Clinician, Interventional Cardiologist.  John D. Symmons, PhD: Basic Scientist

We confirm that we have read this submission and believe that we have an appropriate level of expertise to confirm that it is of an acceptable scientific standard, however we have significant reservations, as outlined above.

# Response

**Published:** 2020-11-30

**Authors:** Yudi Her Oktaviono

**Affiliations:** Department of Cardiology and Vascular Medicine, Faculty of Medicine, University of Airlangga, Soetomo Academic and General Hospital, Indonesia

Dear Dr Anwar Tandar

 Thank you very much for your valuable feedback

 I am truly honoured with your feedback

 In this opportunity, please let me answer the question

* *Q: It is derived from one patient. The conclusion and validity need to be addressed seriously when there is sample from one patient. In addition, since there is only one patient, why is there even a need for inclusion and exclusion criteria.

 A: In this experimental laboratory research, the sample used was EPCs derived from single patient with coronary artery disease. We have ensured the diagnosis of coronary artery disease from the ECG, echocardiography and coronary angiography. The purpose of the single sample being used in this research is to ensure the homogeneity of the sample while inclusion and exclusion criteria was used to identify the best EPCs quality that represent patient with CAD. Mixing EPCs from the various patient may cause EPCs failed to grow efficiently due to incompatibility with other source of EPCs. Inclusion and exclusion criteria is still relevant because we want to ensure that the patient was really diagnosed with coronary artery disease with specific criteria without any other significant disease. Without clear inclusion and exclusion criteria, we may select patient with multiple disease which EPCs function may differ from patient with coronary artery disease only.

 Q: Typographical error in the Introduction... refractory angina, and patients

 A: Thank you for the feedback, we will revise accordingly

**

 Q: Reference needed for the statement: HUCB-MSCs-derived secretome preparation. The media was collected......(reference 3).

 A:  We have added the reference, thank you

*Q: *Treatment of EPCs...Please describe the rationale for 5 replications.  Please describe the rationale behind the selected number 5

 A:  The 5 replication was based on *Federer's formula*: (t-1)(n-1) ≥ 15, where t is the number of treatments and n is the number of replication.

**

*Q: All abbreviations need to be spelt out in the beginning or first time used*

 A: Thank you for the feedback, we will revise accordingly

*Q: *Similar to the rationale for EPCs treatment, in EPC migrations essay section: Please describe the rationale for 5 fields and describe the sizes of the field.

 A: The usage of the 5 fields was the standard protocol of cell migration calculation using transwell migration assay based on the Transwell protocol. In this research, we used 0.04mm in diameter as the sizes of the field.

**

*Q: *This interesting research should investigate more patients to allow a stronger interpretation and to allow more sound conclusion

 A: Thank you very much for your genuine feedback. This research was part of our preliminary research which explore the possibilities of secretome as an alternative regenerative treatment other than cell-based regeneration treatment for the patient with coronary artery disease. In this early research we used limited patient but we will expand further the research with involving more samples  and aim to have clinical trials should we achieve satisfying results. We also consider to combine the secretome treatment with existing coronary artery disease treatment, to ensure the usage of secretome combined with current medical treatment will benefit synergistically.

 Q: Other statins should also be investigated as they are being used widely in real world.

 A: Thank you for the feedback, we will consider that as our future research suggestion. However, we first prefer atorvastatin since it is readily available in our country, Indonesia. Previously, we have compared the effect of atorvastatin, rosuvastatin and simvastatin effect on the EPCs (without secretome) and conclude that atorvastatin was the most superior in inducing EPCs migration. Thus, this research prefers to use atorvastatin and combine it with secretome.

 Q: The exact composition of the hUCB-MSc derived secretome should be described.

 A: Thanks, we would like to admit that the limitation of this research was the inability to exactly describe the molecule in the hUCB-MSc derived secretome. Further research is required through proteomic analysis to determine the molecule in the hUCB-MSc derived secretome. This will also help to standardize the composition of hUCB-MSc derived secretome.

 Again, many thanks for the review,,

 Best Regards

 Yudi Her

# Response

**Published:** 2021-04-22

**Authors:** Yudi Her Oktaviono

**Affiliations:** Department of Cardiology and Vascular Medicine, Faculty of Medicine, University of Airlangga, Soetomo Academic and General Hospital, Indonesia

Dear Dr Anwar Tandar

 Thank you very much for your valuable feedback

 I am truly honoured with your feedback

 In this opportunity, please let me answer the question

* *Q: It is derived from one patient. The conclusion and validity need to be addressed seriously when there is sample from one patient. In addition, since there is only one patient, why is there even a need for inclusion and exclusion criteria.

 A: In this experimental laboratory research, the sample used was EPCs derived from single patient with coronary artery disease. We have ensured the diagnosis of coronary artery disease from the ECG, echocardiography and coronary angiography. The purpose of the single sample being used in this research is to ensure the homogeneity of the sample while inclusion and exclusion criteria was used to identify the best EPCs quality that represent patient with CAD. Mixing EPCs from the various patient may cause EPCs failed to grow efficiently due to incompatibility with other source of EPCs. Inclusion and exclusion criteria is still relevant because we want to ensure that the patient was really diagnosed with coronary artery disease with specific criteria without any other significant disease. Without clear inclusion and exclusion criteria, we may select patient with multiple disease which EPCs function may differ from patient with coronary artery disease only.

 Q: Typographical error in the Introduction... refractory angina, and patients

 A: Thank you for the feedback, we will revise accordingly

**

 Q: Reference needed for the statement: HUCB-MSCs-derived secretome preparation. The media was collected......(reference 3).

 A:  We have added the reference, thank you

*Q: *Treatment of EPCs...Please describe the rationale for 5 replications.  Please describe the rationale behind the selected number 5

 A:  The 5 replication was based on *Federer's formula*: (t-1)(n-1) ≥ 15, where t is the number of treatments and n is the number of replication.

**

*Q: All abbreviations need to be spelt out in the beginning or first time used*

 A: Thank you for the feedback, we will revise accordingly

*Q: *Similar to the rationale for EPCs treatment, in EPC migrations essay section: Please describe the rationale for 5 fields and describe the sizes of the field.

 A: The usage of the 5 fields was the standard protocol of cell migration calculation using transwell migration assay based on the Transwell protocol. In this research, we used 0.04mm in diameter as the sizes of the field.

**

*Q: *This interesting research should investigate more patients to allow a stronger interpretation and to allow more sound conclusion

 A: Thank you very much for your genuine feedback. This research was part of our preliminary research which explore the possibilities of secretome as an alternative regenerative treatment other than cell-based regeneration treatment for the patient with coronary artery disease. In this early research we used limited patient but we will expand further the research with involving more samples  and aim to have clinical trials should we achieve satisfying results. We also consider to combine the secretome treatment with existing coronary artery disease treatment, to ensure the usage of secretome combined with current medical treatment will benefit synergistically.

 Q: Other statins should also be investigated as they are being used widely in real world.

 A: Thank you for the feedback, we will consider that as our future research suggestion. However, we first prefer atorvastatin since it is readily available in our country, Indonesia. Previously, we have compared the effect of atorvastatin, rosuvastatin and simvastatin effect on the EPCs (without secretome) and conclude that atorvastatin was the most superior in inducing EPCs migration. Thus, this research prefers to use atorvastatin and combine it with secretome.

 Q: The exact composition of the hUCB-MSc derived secretome should be described.

 A: Thanks, we would like to admit that the limitation of this research was the inability to exactly describe the molecule in the hUCB-MSc derived secretome. Further research is required through proteomic analysis to determine the molecule in the hUCB-MSc derived secretome. This will also help to standardize the composition of hUCB-MSc derived secretome.

 Again, many thanks for the review,,

 Best Regards

 Yudi Her

# Peer review: Reviewer response for version 1

**DOI:** 10.5256/f1000research.25984.r64524

**Published:** 2020-07-08

**Authors:** Anwar Santoso

**Affiliations:** 1 Department of Cardiology, Faculty of Medicine, University of Indonesia, Jakarta, Indonesia; 2 National Cardiovascular Centre, Harapan Kita Hospital, Jakarta, Indonesia

- The study aims to compare the effect of hUCB-MSC-derived secretome, atorvastatin, and the two combinations in modulating EPC proliferation and migration. The study addresses the novel issues in refractory angina, whether the atorvastatin and secretome derived mesenchymal stem cells improve EPC expression. There are similar studies available addressing these issues. Zhang X et al. demonstrated that intravenous transplantation of huC-MSCs at an early stage could improve hypoxic-ischemic rats' behavior and decreased gliosis, this study was measured in other target disorders ^1^.
- The rationale and scientific background of this manuscript were justified, to disclose the role of secretomes and paracrine stimulation on EPC expression.
- In the #Method section, the authors mentioned the inclusion criteria: male, aged 40-59 years old, history of chronic ischemic heart disease as proven by CAG (coronary angiography). The authors *should quote the diagnostic criterion*. Additionally, the authors *should clearly state this is an experimental study* of atorvastatin and hUCB-MSC-derived secretome and their combinations on EPC proliferation and migration. 
- Umbilical cord blood-derived EPC established in a previous study displayed *cobblestone-like morphology*; this is a typical feature of the EPC. The authors did not state this in the manuscript, except they confirmed using FITC-labelled anti-human CD 34+ expression. *The authors should clearly explain it[ref-*2].
- There was another immunophenotype of EPC as determined by flow cytometry, VEGFR2-PE, vWF-FITC, and CD31-PE ^2^. Is there any reason why the authors only demonstrated with CD34+ expression. *The authors should provide their ideas on it*. 
- In the #EPCs proliferation assay, the authors explain EPCs proliferation measured using OD; there was no explanation of how OD transferred in a measurement scale in Figure 2 (Y-ordinate)?
- How did the authors determine the percentage of hUCB-MSC-derived secretome? Is there any control over the measurement?
- In the #Table 1 – Characteristics of the patient, the authors should explain "Left ventricle internal diameter" = 5.8 cm; I wonder whether that is either "end-systolic dimension or end-diastolic dimension"? 
- In #summary, the authors should open the opportunity on the horizon, whether the cell-based therapy or cell-free measures that win the future game? ^3^.

Again, I would express my appreciation to all authors to address these evolving issues in regenerative medicine.

Is the work clearly and accurately presented and does it cite the current literature?

Yes

If applicable, is the statistical analysis and its interpretation appropriate?

Yes

Are all the source data underlying the results available to ensure full reproducibility?

Partly

Is the study design appropriate and is the work technically sound?

Yes

Are the conclusions drawn adequately supported by the results?

Yes

Are sufficient details of methods and analysis provided to allow replication by others?

Partly

Reviewer Expertise:

1. Cardiovascular disease (particularly ischemic heart disease) 2. Lipidology and diabetes mellitus 3. Hypertension 4. Stem cell and regenerative medicine

I confirm that I have read this submission and believe that I have an appropriate level of expertise to confirm that it is of an acceptable scientific standard, however I have significant reservations, as outlined above.

## References

[1] : Therapeutic effect of human umbilical cord mesenchymal stem cells on neonatal rat hypoxic-ischemic encephalopathy.*J Neurosci Res*.2014;92(1) : 10.1002/jnr.2330435-4510.1002/jnr.2330424265136

[2] : Endothelial Progenitor Cell Migration-Enhancing Factors in the Secretome of Placental-Derived Mesenchymal Stem Cells.*Stem Cells Int*.2016;2016: 10.1155/2016/2514326251432610.1155/2016/251432626880942PMC4736766

[3] : Mechanisms of Cardiac Repair and Regeneration. *Circulation Research*.2018;122(8) : 10.1161/CIRCRESAHA.117.3125861151-116310.1161/CIRCRESAHA.117.312586PMC619104329650632

# Response

**Published:** 2020-08-16

**Authors:** Yudi Her Oktaviono

**Affiliations:** Department of Cardiology and Vascular Medicine, Faculty of Medicine, University of Airlangga, Soetomo Academic and General Hospital, Indonesia

1.

 Q The study aims to compare the effect of hUCB-MSC-derived secretome, atorvastatin, and the two combinations in modulating EPC proliferation and migration. The study addresses the novel issues in refractory angina, whether the atorvastatin and secretome derived mesenchymal stem cells improve EPC expression. There are similar studies available addressing these issues. Zhang X et al. demonstrated that intravenous transplantation of huC-MSCs at an early stage could improve hypoxic-ischemic rats' behavior and decreased gliosis, this study was measured in other target disorders ^1^.

 A. Thank you for the references and comparison, we will note this feedback and put it on the paper as other research which did similar intervention

 2.

 Q. The rationale and scientific background of this manuscript were justified, to disclose the role of secretomes and paracrine stimulation on EPC expression.I

 A. ndeed, that was our research aim

 3.

 Q. In the #Method section, the authors mentioned the inclusion criteria: male, aged 40-59 years old, history of chronic ischemic heart disease as proven by CAG (coronary angiography). The authors *should quote the diagnostic criterion*. Additionally, the authors *should clearly state this is an experimental study* of atorvastatin and hUCB-MSC-derived secretome and their combinations on EPC proliferation and migration. 

 A. We have added the diagnostic criteria of chronic ischemic heart disease as proven by coronary angiography results that showed >50% stenosis of left main coronary artery or >70% of other coronary arteries. We also explain that *this is an experimental study* of atorvastatin and hUCB-MSC-derived secretome and their combinations on EPC proliferation and migration. 

  4.

 Q. Umbilical cord blood-derived EPC established in a previous study displayed *cobblestone-like morphology*; this is a typical feature of the EPC. The authors did not state this in the manuscript, except they confirmed using FITC-labelled anti-human CD 34+ expression. *The authors should clearly explain it[ref-*2].

 A. Thank you very much for reminding us, We did evaluate the EPCs cobblestone-like morphology before staining the cells with CD34 antibody. We already put these information on the method section.

 5. 

 Q. There was another immunophenotype of EPC as determined by flow cytometry, VEGFR2-PE, vWF-FITC, and CD31-PE ^2^. Is there any reason why the authors only demonstrated with CD34+ expression. *The authors should provide their ideas on it*. 

 A. In this study, the use of FITC CD34+ only to confirmed the EPCs are sufficient, as the same EPCs culture method was used in authors previous research ( which mentioned on references number 19, 21-22,45). There was also uncertainty about the use of another immunophenotype of EPC as determined by flow cytometry (VEGFR2-PE, vWF-FITC, and CD31-PE), caused by heterogenous types of EPCs (mentioned on the references of 51,52)

 6. 

 Q. In the #EPCs proliferation assay, the authors explain EPCs proliferation measured using OD; there was no explanation of how OD transferred in a measurement scale in Figure 2 (Y-ordinate)?

 A. EPCs proliferation can be determined through various method, In the previous study, proliferation can be determined relatively through OD (references no 21-22,45). Thus, we use OD  as proliferation measurement scale in Figure 2 (Y-ordinate).

 7. 

 Q.How did the authors determine the percentage of hUCB-MSC-derived secretome? Is there any control over the measurement?

 A. The percentage of hUCB-MSC-derived secretome was determined from the dilution level of hUCB-MSC-derived secretome. For example, 1 mL of hUCB-MSC-derived secretome with 49 mL of the phosphate buffer saline (PBS) plus 2% fetal bovine serum (FBS). However, no control over measurement.

 8.

 Q. In the #Table 1 – Characteristics of the patient, the authors should explain "Left ventricle internal diameter" = 5.8 cm; I wonder whether that is either "end-systolic dimension or end-diastolic dimension"? 

 A. It should be Left ventricle end-diastolic dimension, thanks for correcting this phrase

 9.

 Q. In #summary, the authors should open the opportunity on the horizon, whether the cell-based therapy or cell-free measures that win the future game?

 A. Cardiac cell-based therapy has emerged as a novel therapeutic option for patients dealing with untreatable refractory angina (RA). However, after more than a decade of controlled studies, no definitive consensus has been reached regarding clinical efficacy. While in this research hUCB-MSC-derived secretome may be developed and combined with atorvastatin treatment in CAD patients to improve EPCs proliferation and migration. In this early study, we can conclude that secretome-based treatment may be a game changer in refractory angina therapeutic options and outperforms the previous cell-based therapy.

 Again, I would express my appreciation to all authors to address these evolving issues in regenerative medicine.

# Response

**Published:** 2021-04-22

**Authors:** Yudi Her Oktaviono

**Affiliations:** Department of Cardiology and Vascular Medicine, Faculty of Medicine, University of Airlangga, Soetomo Academic and General Hospital, Indonesia

1.

 Q The study aims to compare the effect of hUCB-MSC-derived secretome, atorvastatin, and the two combinations in modulating EPC proliferation and migration. The study addresses the novel issues in refractory angina, whether the atorvastatin and secretome derived mesenchymal stem cells improve EPC expression. There are similar studies available addressing these issues. Zhang X et al. demonstrated that intravenous transplantation of huC-MSCs at an early stage could improve hypoxic-ischemic rats' behaviour and decreased gliosis, this study was measured in other target disorders ^1^.

 A. Thank you for the references and comparison, we will note this feedback and put it on the paper as for other research which did a similar intervention

 2.

 Q. The rationale and scientific background of this manuscript were justified, to disclose the role of secretomes and paracrine stimulation on EPC expression

 A. Indeed, that was our research aim

 3.

 Q. In the #Method section, the authors mentioned the inclusion criteria: male, aged 40-59 years old, history of chronic ischemic heart disease as proven by CAG (coronary angiography). The authors *should quote the diagnostic criterion*. Additionally, the authors *should clearly state this is an experimental study* of atorvastatin and hUCB-MSC-derived secretome and their combinations on EPC proliferation and migration. 

 A. We have added the diagnostic criteria of chronic ischemic heart disease as proven by coronary angiography results that showed >50% stenosis of left main coronary artery or >70% of other coronary arteries. We also explain that *this is an experimental study* of atorvastatin and hUCB-MSC-derived secretome and their combinations on EPC proliferation and migration. 

  4.

 Q. Umbilical cord blood-derived EPC established in a previous study displayed *cobblestone-like morphology*; this is a typical feature of the EPC. The authors did not state this in the manuscript, except they confirmed using FITC-labelled anti-human CD 34+ expression. *The authors should clearly explain it[ref-*2].

 A. Thank you very much for reminding us, We did evaluate the EPCs cobblestone-like morphology before staining the cells with CD34 antibody. We already put these information on the method section.

 5. 

 Q. There was another immunophenotype of EPC as determined by flow cytometry, VEGFR2-PE, vWF-FITC, and CD31-PE ^2^. Is there any reason why the authors only demonstrated with CD34+ expression. *The authors should provide their ideas on it*. 

 A. In this study, the use of FITC CD34+ only to confirmed the EPCs are sufficient, as the same EPCs culture method was used in authors previous research ( which mentioned on references number 19, 21-22,45). There was also uncertainty about the use of another immunophenotype of EPC as determined by flow cytometry (VEGFR2-PE, vWF-FITC, and CD31-PE), caused by heterogenous types of EPCs (mentioned on the references of 51,52)

 6. 

 Q. In the #EPCs proliferation assay, the authors explain EPCs proliferation measured using OD; there was no explanation of how OD transferred in a measurement scale in Figure 2 (Y-ordinate)?

 A. EPCs proliferation can be determined through various method, In the previous study, proliferation can be determined relatively through OD (references no 21-22,45). Thus, we use OD  as proliferation measurement scale in Figure 2 (Y-ordinate).

 7. 

 Q.How did the authors determine the percentage of hUCB-MSC-derived secretome? Is there any control over the measurement?

 A. The percentage of hUCB-MSC-derived secretome was determined from the dilution level of hUCB-MSC-derived secretome. For example, 1 mL of hUCB-MSC-derived secretome with 49 mL of the phosphate buffer saline (PBS) plus 2% fetal bovine serum (FBS). However, no control over measurement.

 8.

 Q. In the #Table 1 – Characteristics of the patient, the authors should explain "Left ventricle internal diameter" = 5.8 cm; I wonder whether that is either "end-systolic dimension or end-diastolic dimension"? 

 A. It should be Left ventricle end-diastolic dimension, thanks for correcting this phrase

 9.

 Q. In #summary, the authors should open the opportunity on the horizon, whether the cell-based therapy or cell-free measures that win the future game?

 A. Cardiac cell-based therapy has emerged as a novel therapeutic option for patients dealing with untreatable refractory angina (RA). However, after more than a decade of controlled studies, no definitive consensus has been reached regarding clinical efficacy. While in this research hUCB-MSC-derived secretome may be developed and combined with atorvastatin treatment in CAD patients to improve EPCs proliferation and migration. In this early study, we can conclude that secretome-based treatment may be a game changer in refractory angina therapeutic options and outperforms the previous cell-based therapy.

 Again, I would express my appreciation to all authors to address these evolving issues in regenerative medicine.
